# Efficient Genome Editing in Apple Using a CRISPR/Cas9 system

**DOI:** 10.1038/srep31481

**Published:** 2016-08-17

**Authors:** Chikako Nishitani, Narumi Hirai, Sadao Komori, Masato Wada, Kazuma Okada, Keishi Osakabe, Toshiya Yamamoto, Yuriko Osakabe

**Affiliations:** 1 NARO Institute of Fruit Tree and Tea Science, 2-1 Fujimoto, Tsukuba, Ibaraki 305-8605, Japan; 2 Faculty of Agriculture, Iwate University, 3-18-8 Ueda, Morioka, Iwate 020-8550, Japan; 3 NARO Institute of Fruit Tree and Tea Science, 92-24, Nabeyashiki, Shimokuriyagawa, Morioka, Iwate 020-0123, Japan; 4 Faculty of Bioscience and Bioindustry, Tokushima University, 2-1 Josanjima, Tokushima 770-8513, Japan

## Abstract

Genome editing is a powerful technique for genome modification in molecular research and crop breeding, and has the great advantage of imparting novel desired traits to genetic resources. However, the genome editing of fruit tree plantlets remains to be established. In this study, we describe induction of a targeted gene mutation in the endogenous apple phytoene desaturase (*PDS*) gene using the CRISPR/Cas9 system. Four guide RNAs (gRNAs) were designed and stably transformed with Cas9 separately in apple. Clear and partial albino phenotypes were observed in 31.8% of regenerated plantlets for one gRNA, and bi-allelic mutations in apple *PDS* were confirmed by DNA sequencing. In addition, an 18-bp gRNA also induced a targeted mutation. These CRIPSR/Cas9 induced-mutations in the apple genome suggest activation of the NHEJ pathway, but with some involvement also of the HR pathway. Our results demonstrate that genome editing can be practically applied to modify the apple genome.

Apple is one of the main fruit crops in temperate regions of the world. Global commercial apple production exceeded 80 Mt in 2013 (FAOSTAT, http://faostat.fao.org), and various apple cultivars with superior quality have been developed. Since fruit trees typically have long breeding cycles, it takes effort to produce commercial varieties with the desired phenotypic traits, especially given the poor genetic resources available for traditional breeding. Furthermore, most fruit trees are heterozygotes and are produced by clonal propagation, and, consequently, backcross breeding to transfer specific traits is difficult. The recent release of the apple genome sequence^1^ has produced major advances in apple genetics. Genome sequences can now be used to identify the genes controlling important traits, and to plan specific modification of targeted genes in apple.

Genome editing technologies using engineered nucleases have been developed as effective genetic engineering methods to target and digest DNA at specific locations in the genome^2^. To date, three main types of engineered nucleases have been developed for genome editing: zinc finger nuclease (ZFN), transcription activator-like effector nuclease (TALEN), and CRISPR (clustered regularly interspaced short palindromic repeats)/Cas9. Among these genome editing approaches, due to its simplicity, design flexibility, and high efficiency, the CRISPR/Cas9 system is now utilized widely for editing the genome of various organisms of all types, including plants, animals, yeast and bacteria. In the CRISPR/Cas9 system, Cas9 endonuclease—an RNA-guided nuclease from *Streptococcus pyogenes*—can be engineered to target specific DNA sequences using recognition via complementary base pairing to a guide RNA (gRNA). Following DNA digestion, two major repair pathways—homologous recombination (HR) and non-homologous end-joining (NHEJ)—are activated. Since the HR pathway repairs the digested DNA precisely using templates, it can be used in gene targeting. On the other hand, in the NHEJ pathway, the ends of the digested DNA are simply connected, and base deletion/insertion, or replacement, is induced, resulting in the disruption of gene function. Both the NHEJ and HR pathways have been demonstrated to operate in various plant species^3^. It is suggested that the NHEJ pathway also functions in *populus*^4^, in which high mutation rates were obtained by using four gRNAs simultaneously. A practical method of efficient targeted mutation is especially important for clonally propagated plants because it enables selection of homozygous plants or release of their chimeric nature.

Here, we present the first study to show efficient apple genome editing using a single gRNA and Cas9 in first generation transgenic apple. Using this system, an apple phytoene desaturase (*PDS*) gene was precisely modified. Among the transformants derived from one of the four designed gRNAs, 13.6% had a clear albino phenotype, and bi-allelic targeted mutagenesis was confirmed by DNA analysis. In addition, an 18-bp gRNA also induced mutation in the apple *PDS* gene in apple. To our knowledge, this is the first report of apple genome editing, and the findings presented here will contribute to the application of genome editing methodologies to apple breeding programmes.

## Results

### Cloning of the apple *PDS* gene

**Figure 1:**
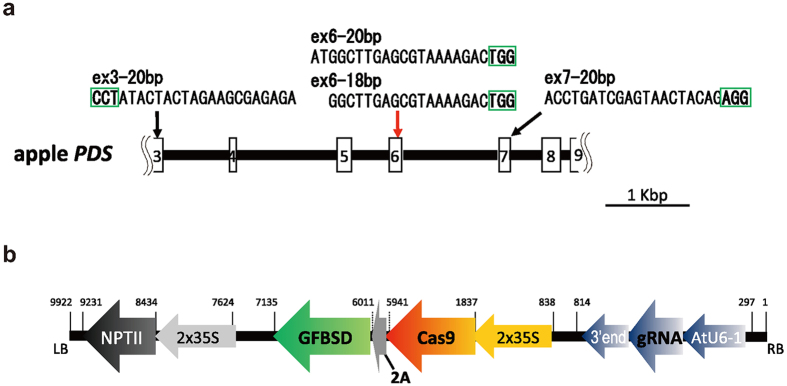
Schematic diagram of CRISPR/Cas9 target sites in the apple *PDS* gene, and vector construction. (**a**) Schematic position of four guide RNAs (gRNAs) targeting the apple *PDS* genome sequence. Boxes indicate exons; numbers indicate exon numbers; lines indicate introns. The sequence of each gRNAs is shown, the PAM sequences are surrounded by green boxes. (**b**) Schematic map of the CRISPR/Cas9 vector for plant stable transformation mediated by Agrobacterium. Numbers indicate the position in the vector, the right border is numbered 1–26.

In order to test whether the CRISPR/Cas9 system can induce mutation effectively in the apple genome, we selected the apple phytoene desaturase (*PDS*) gene, which is required for chlorophyll biosynthesis^5^ as a target gene. In other plant species, *pds* mutant plants show an albino phenotype^5^, and have served as a model for CRISPR/Cas9-mediated targeted gene editing^4^,^6^,^7^,^8^. A 4801-bp fragment corresponding to the partial apple *PDS* genome region was cloned (accession no. LC101839) from rootstock cultivar ‘JM2’. Comparison of reported apple *PDS* ESTs and *PDS* ESTs from other plant species showed that the cloned region spanned from the middle of the third exon to the middle of the ninth exon of the apple *PDS* gene (Fig. 1a). According to the draft apple genome sequence^1^, the apple *PDS* gene is considered a single gene.

### Constructs for targeted genome editing of the apple *PDS* gene

To introduce mutations into the apple *PDS* gene, we designed gRNAs as target sites for CRIPSR/Cas9. Three 20-bp sequences with a tri-nucleotide 5′-NGG-3′, the protospacer-adjacent motif (PAM), on their 3´-regions in the third, sixth and seventh exon of the apple *PDS* genome sequence, were selected as target sites, and were named ex3–20 bp, ex6–20 bp and ex7–20 bp, respectively (Fig. 1a). These sequences were selected by *in silico* analysis using the CasOT program to avoid off-target effects^9^. In addition, we also selected a truncated 18-bp sequence as the gRNA for the sixth exon (ex6-18 bp, Fig. 1a), based on recent findings showing that truncated gRNAs can induce mutations in mammalian cells with low off-target effects^10^. Taking into consideration that apple genomes are highly heterogeneous^1^, we then confirmed that no SNPs were detected at the target sites, although some polymorphisms surrounding the target sites were found (Supplementary Fig. S1). Each of the four target sequences was inserted into a gRNA expression cassette together with the *Cas9* endonuclease coding sequence in the all-in-one plant binary vector pEgP226-2A-gfbsd2 (Fig. 1b)^11^, which harbors a plant codon-optimized Cas9 fused to *GFBSD2* (a fusion gene of GFP and brastcidin resistance gene via the 2A peptide)^11^,^12^ under the 2 × 35SCaMV promoter, and a gRNA expression cassette under the *AtU6-1* promoter (Fig. 1b). Thus, GFP expression can be used to monitor and select Cas9-expressing cells using this vector^11^. For this study, we used the kanamycin-resistance cassette in the vector for selection of apple transformants. All four constructs were transformed to apple ‘JM2’ leaf discs separately using the agrobacterium method.

### Phenotypic analysis of CRISPR/Cas9 transgenic apple lines

**Figure 2:**
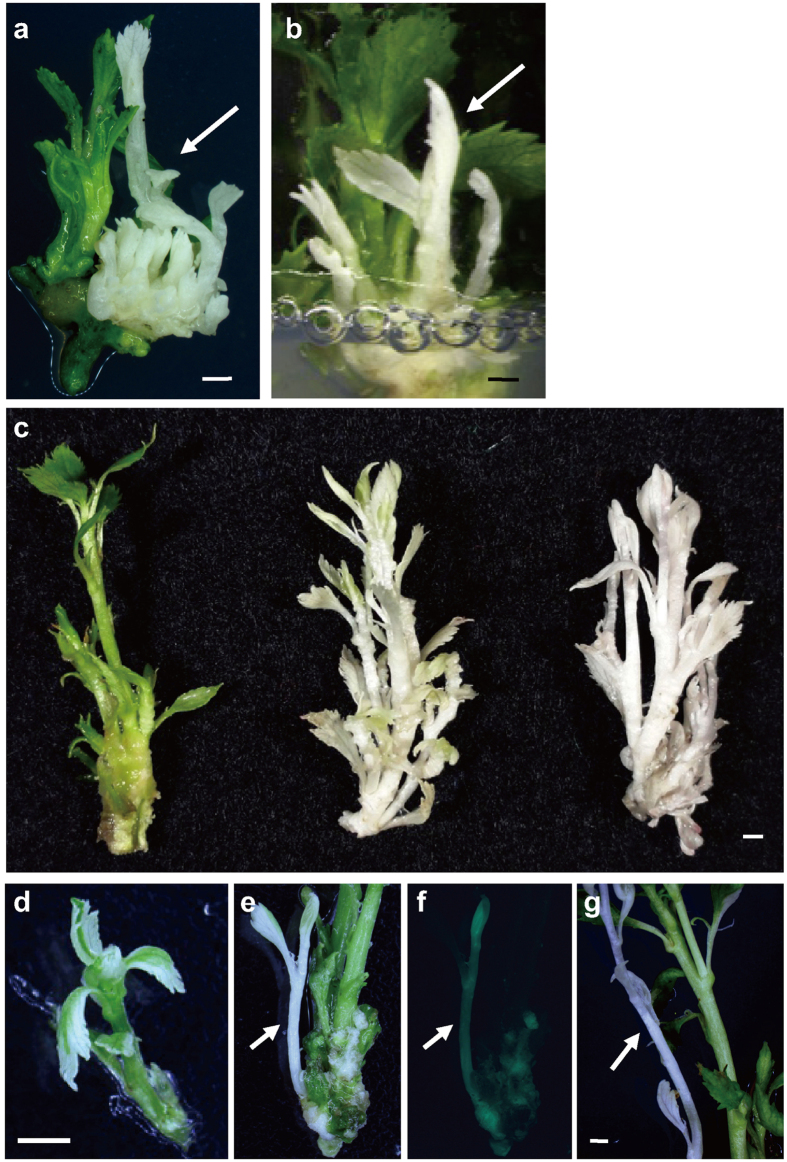
Phenotypes of CRIPSR/Cas9-induced mutant apples. (**a**,**b**) Clear albino transformants, ex7–20 bp#89A and ex7–20 bp#49A. Arrows indicate albino transformants regenerated around 8 months after transformation. The green shoots are transformants without visible phenotype. (**c**) Left; wild type ‘JM2’ shoot. Middle; pale green transformant with chimeric mutations, ex7–20 bp#70B. Right; pale green transformant regenerated from the same leaf disk as ex7–20 bp#70B. (**d**) A typical variegated transformant with chimeric mutations, ex7–20 bp#49C. The most variegated transformant had leaves edged in white to pale green while the central part is green. (**e**) Pale green transformant targeted on third exon, ex3–20 bp#81B. (**f**) GFP fluorescence of (**e**). (**g**) Albino transformant, ex6-18bp#54. The green shoots are transformants without visible phenotypes. Bars: 1 mm.

Transgenic apple shoots with visible mutant phenotypes, e.g., albino (Fig. 2a,b,g), pale green [Fig. 2c (middle and right), E] and variegated (Fig. 2d), were regenerated from the basal region of green shoots around 8 months after transformation. The albino phenotypes in the transgenic apples were similar to those seen in *pds* mutants in other plants, suggesting that the CRIPSR/Cas9 targeting of the apple *PDS* induced the mutation. In some cases, various types of mutated shoots regenerated simultaneously from the same leaf discs, although it remains possible that transformants regenerated from the same leaf disc were not independent clones. We therefore classified the leaf discs used for the transformation into three groups according to the phenotypes of the transgenic T_0_ shoots regenerated from them as follows (Table 1): (1) clear albino transformants (with pale green or variegated shoots), (2) at least pale green or variegated without albino sectors, and (3) exclusively green transformants. Table 1 shows the number of mutant plants for each gRNA; it can be seen that the most efficient gRNA, inducing the strongest phenotype, was ex7–20 bp (Table 1), for which mutants with visible phenotypes were generated with high efficiency. A pale green transformant was also observed for ex3–20 bp with GFP fluorescence (Fig. 2e,f), and a clear albino plant was obtained by using the truncated gRNA, ex6–18 bp (Fig. 2g). On the contrary, no visible mutant phenotypes were detected in any of the ex6-20bp transformants.

### Targeted mutation of the apple *PDS* gene by CRIPSR/Cas9

**Figure 3:**
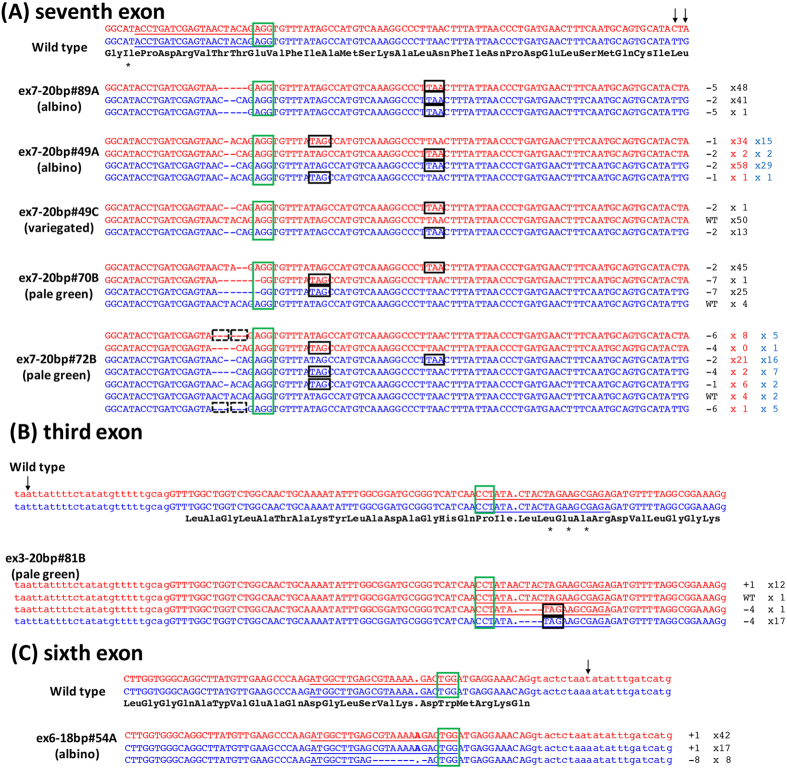
Mutations in the apple *PDS* gene induced by CRIPSR/Cas9. (**A**) The apple *PDS* genome sequences surrounding the ex7–20 bp target site. The clear albino plants, ex7–20 bp#89A and #49A, corresponding to the plants shown by arrows in Fig. 2a,b respectively. ex7–20 bp#49C and #70B correspond to the plants in Fig. 2d and the middle of Fig. 2c, respectively. (**B**) The apple *PDS* sequences surrounding the ex3-20bp target site. ex3-20bp#81B corresponds to the plant in Fig. 2e. (**C**) The apple *PDS* sequences surrounding the ex6–18 bp target site. ex6-18bp#54A corresponds to the plant in Fig. 2g. Top, the apple *PDS* genome sequences of wild-type ‘JM2’. Arrowheads indicate the single nucleotide polymorphisms distinguishing each allele, with one allele written in red and the other in blue. The target sequences are underlined and PAM sequences are in green boxes. The predicted amino acids are shown under the wild-type sequences. Upper case letters; exon sequences, lower case letters; intron sequences. The phenotypes of transformants are noted in parenthesis. The right side numbers indicate the number of inserted (+) and deleted (−) bases and detected clone numbers in the sequence analysis. For ex7–20 bp#49A and ex7–20 bp#72B, two primer sets were used, the clone numbers obtained from 4561F-26 bp and 4704R-27 bp(A) (red) are on the left, and those from 4561F-26 bp and 4704R-27 bp(G) are on the right (blue). Boxes with solid black lines indicate the stop codons formed by the mutations. Boxes with dotted line indicate the deletion of amino acid(s). The Ile (seventh exon) and Leu-Glu-Ala (third exon) with asterisks are predicted as functionally important amino acids.

To verify the mutated sequences at the target sites in transformants induced by CRISPR/Cas9, genomic sequences isolated from T_0_ apple shoots were analyzed. Two types of sequence were amplified by PCR using primers designed to amplify about 200–500 bp surrounding each target site (Fig. 3, Supplementary Fig. S1). Since the apple *PDS* gene is predicted to be a single-copy gene based on the genome sequence of apple^1^, distinguishing sequences were concluded to represent allele polymorphism. The genome regions around the targets were amplified by PCR and cloned, and more than 30 clones for each transformant with a visible phenotype (more than 54 clones for each clear albino transformant) were randomly selected and sequenced. The remaining transformants were analyzed by direct PCR to detect partial mutations. The PCR results revealed that all transformants with visible phenotypes showed mutated sequences in the apple *PDS* gene. Two green transformants with no visible altered phenotype were found to have partially mutated sequences (Table 1).

From the above sequence analysis, the gRNA target resulting in the most efficient mutation was ex7–20 bp. All the clear albino ex7–20 bp transformants contained only the mutated allele sequence at a position 3–4 bases upstream of the PAM sequence in the target site (Fig. 3A). Almost all alleles analyzed had two types of mutated sequences, which were classified into major and minor mutations. A typical example was the genome sequence of ex7–20 bp #49A, whose clear albino transformants had a total of 49 clones with a 1-base deletion and four clones with a 2-base deletion for one allele, as revealed by PCR using two types of primers for each allele (Fig. 3A, Supplementary Fig. S1). Another allele of the ex7–20 bp #49A mainly had the same 2-base deletion mutation, and, as the minor mutation, a 1-base deletion that was the same as the major mutation of another allele (Fig. 3A). Similarly, in almost all the clear albino transformants analyzed by the two primer sets, the minor sequences in one allele were detected as the major sequence in another allele (Fig. 3A). In the variegated or pale green transformants, not only mutated but also wild-type sequences were detected (Fig. 3A), and sometimes more than three types of mutated sequences were detected in one allele (ex7–20 bp#72B, Fig. 3A). In some cases, transformants with different phenotypes regenerated from identical leaf discs showed the same mutated sequence as predicted above. This was typically exemplified by ex7–20 bp lines #49A and #49C, which were regenerated from the same leaf disc and shared the same 2-bp deletion mutation (Fig. 3A). That the T-DNA insertion sites in these lines were identical was demonstrated by TAIL-PCR (Supplementary Fig. S2). Both the wild-type and mutated sequences were obtained from pale green transformants targeted by ex3–20 bp (Fig. 3B). In the case of a clear albino transformant targeted by ex6–18 bp, a 1-base insertion and an 8-base deletion were obtained (Fig. 3C). The same mutated sequences between the alleles were also found in the third and sixth exon.

The results of sequence analysis indicated that all the apple *PDS* mutant alleles were insertion or deletions (+1 to –8 bases) regardless of target site. The mutations corresponding to 1-base insertion and 1-, 2-, 4-, 5-, 7- and 8-base deletions generated frameshift mutations and stop codons near the target site, leading to truncated PDS protein. For example, a 1-base insertion mutation in ex3–20 bp#81B generated an ectopic stop codon in the fourth exon (836 bp downstream of the mutated site) (Fig. 3B), and a 1-base insertion and 8-base deletion in ex6–18 bp#54A led to an ectopic stop codon in the seventh exon (1160 bp downstream of the mutated site) (Fig. 3C). Mutations of 3- and 6-base deletions mediated by ex7–20 bp resulted in deletion of 1 or 2 amino acids near the isoleucine in a functionally important region of PDS^13^ (Fig. 3). Thus, the sequence data confirmed the loss of function of the apple *PDS* gene caused by the intended targeted mutations.

## Discussion

To apply genome editing in apple, we used CRISPR/Cas9 to target the endogenous apple *PDS* gene using a single gRNA. This is the first report of genome editing in Rosaceae—one of the major plant families, to which various important fruit and flower species belong. Our study suggests that mutations were induced successfully and efficiently in the apple genome within 8 months, which is generally a shorter time period than that required for traditional mutation breeding. Recent reports described induction of mutations in mammalian cells with low off-target effects using an 18-bp gRNA^10^. An 18-bp gRNA was also able to induce targeted genome editing in apple in the present study. Off-target mutation should be further analyzed.

The efficiency of genome editing in apple depended on the target site sequence. It should be noted that the 13.6% (6 out of 44 leaf discs generated clear albino transformants) of the apple plantlets corresponded to almost perfect mutation in the first generation. The generation of homozygous knockout mutations is very important for genetic modification of trees with a long life span and genomic heterozygosity. Our study showed that the efficiency was high enough to induce the homozygous knockout mutation of a *PDS* gene in apple. Apple plants with a visible albino phenotype showed relatively slow growth rates compared to those without visible phenotypes. Further analysis is needed to investigate whether this is due to the effect of apple *PDS* destruction or to the nature of apple genome editing.

Variegated mutants were also obtained with several gRNAs. In most of the variegated apple transformants, the edges of the leaves were white or pale green. The results suggest preferential induction of these mutations in the L2 layer in our system based on the observation that almost all mesophyll tissues at the margin of the leaf blade are of L2 origin^14^,^15^. When aiming to modify fruit traits, it is desirable to induce mutation of a L2 layer in the cell lineage, because fruit tissues originate mainly from the L2 layer^15^. On the other hand, the chimeric release of variegated mutants is needed to stabilize the desired traits. In our study, apple shoots containing large amounts of mutated cells tended to be formed by ramification of green shoots mutated at a lower rate, suggesting the culture conditions promoting the ramification might be effective in ensuring chimeric release. Indeed, transformants with various phenotypes regenerated from the same leaf discs shared the same mutation and the same T-DNA insertion site(s) (Supplementary Fig. S2). The results suggested that the same mutations in transformants from the same leaf discs arose because these transformants were from the same cell lines.

All the mutations detected in apple were short insertions or deletions, as a consequence of repair via non-homologous end joining (NHEJ) following gRNA-directed Cas9 cleavage, similar to the case in *populus*^4^. Analysis of alleles distinguished by their sequence polymorphism suggested activation also of the HR pathway in apple. Notably, the minor types of mutated sequence were sometimes related to the major types of mutated sequence of another allele, so might have been generated though a copying mechanism. This hypothesis should be analyzed further; however, if the HR pathway is activated in apple as well as the NHEJ pathway, gene targeting with high efficiency to replace genes with desirable alleles would be possible.

At the present stage of development, apple genome editing is applicable to the study of gene function and to finding novel important genes by constructing apple mutant libraries. So far, molecular and physiological analysis have supplied information on genes related to some important apple traits. For example, self-incompatibility (SI) mechanisms, which work in apple and in a wide range of other species to prevent self-fertilization, are controlled by a single S-locus^16^. It has been shown that the SI S alleles of the Rosaceae, including apples, encode members of a T2/S ribonuclease superfamily (S-RNase)^17^, and indeed self-fertile apples were obtained by S-RNase gene silencing^18^. In addition, polyphenol oxidase is responsible for apple flesh browning^19^, putative 2OG-Fe(II) oxygenase regulates columnar habit in apple^20^, and the Myb transcription factor regulates red coloration in apple fruits^21^. These findings provide useful knowledge for the future alteration of gene sequences to impart desired apple traits.

For application to apple breeding, foreign-gene-free techniques will be needed. To date, several methods, for example, the use of *piggyBac* transposon, have been developed successfully to remove foreign footprints from rice transformants^22^. Furthermore, the direct delivery of Cas9 protein and appropriate gRNAs into plant cells^23^, development of the delivery system itself^24^, efforts to improve the regeneration ratio from apple explants^25^, and the combination of these techniques could enable us to obtain apple with edited genomes without a transformation process. Together with improvements in mutation efficiency and gene targeting using CRISPR/Cas9, such systems would contribute to further molecular breeding to generate desired apple traits.

## Materials and Methods

### Growth and transformation of apple

The apple semi-dwarfing rootstock cultivar ‘JM2’ (*Malus prunifolia* (Wild.) Borkh. ‘Seishi’ × *M. pumila* Mill. var. *paradisiaca* Schneid. ‘M.9’) was cultured *in vitro* and the leaflets were used in transformation experiments as described previously^26^. Genomic DNA was extracted from stable transgenic and wild-type plants using DNeasy (Qiagen, Hilden, Germany) according to the manufacturer’s protocol. To confirm agrobacterium elimination in regenerated plantlets with kanamycin resistance, genomic DNA was amplified by PCR using agrobacterium-specific primers (VCF: 5′-ATCATTTGTAGCGACT-3′, VCR: 5′-AGCTCAAACCTGCTTC-3′)^27^. Plantlets showing no PCR amplification product were selected as “agrobacterium eliminated” and used for further analysis. To confirm the transformation of *gRNA*, *Cas9* and *NPTII* genes, genomic DNA was amplified in separate PCR assays. The gRNA cassette specific primers were AtU6-1proF297-314 (5′-TTCCGTGGGAGAAATCTC-3′) and AtU6-3endR814-796 (5′-TTCGCGCAGATTTGCATCC-3′). The primers for *Cas9* were Cas9F5470-5499 (5′-CCTCCCTTCCAAGTACGTCA-3′) and Cas9R5734-5715 (5′-GAGGGTGAAGAGGTGGATGA-3′). And the primers for *NPTII* gene were NPTIIF8434-8453 (5′-ATGGGGATTGAACAAGATGG-3′) and NPTIIR9230-9210 (5′-CAGAAGAACTCGTCAAGAAG-3′). The PCR reaction was carried out with GoTaq DNA polymerase (Promega, Japan) in a total volume of 10 μL at 94 °C for 5 min; 35 cycles of 94 °C for 1 min, 50 °C for 1 min and 72 °C for 2 min, followed by a final extension of 72 °C for 7 min.

### Cloning of an apple *PDS* gene

The apple ‘JM2’ genomic DNA fragment of the *PDS* gene was amplified by PCR with the gene-specific primers (apple PDS-F: 5′-TTRTCWACWGCAAARTAYYTGG-3′; apple PDS-R: 5′-TTACCATATGTGAACATTGATAACTGG-3′) designed based on its homologous gene (MDP0000148978) sequence in *M. domestica* and the conserved region of *PDS* ESTs from other species; arabidopsis (L16237), tomato (X59948), pepper (X68058), soy bean (M64704). The PCR reaction was carried out with ExTaq DNA polymerase (Takara, Japan) in a total volume of 100 μL at 94 °C for 3 min; 35 cycles of 94 °C for 1 min, 50 °C for 1 min and 72 °C for 5 min, followed by a final extension of 72 °C for 10 min. The PCR product was cloned and sequenced. For analysis of intron-exon structure, the sequence was compared with reported apple *PDS* ESTs (accession no. GO517828.1, GO499218.1, GO523095.1, GO514137.1, GO547261.1) and *PDS* ESTs from other plant species shown above.

### Construction of CRISPR/Cas9 vectors

For construction of all-in-one CRISPR/Cas9 vectors, a plant codon-optimized (GC-rich version) *Streptococcus pyogenes Cas9*, namely “*fcoCas9*”, fused to *GFBSD2*^11^,^12^ via a self-cleaving 2A peptide inserted between the CaMV35SΩ promoter and the *hsp18.2* terminator^28^ in the binary vector pCAMBIA. In these constructs, a FLAG-tag and NLSs were fused to the N-terminal end of fcoCas9, with a NLS also fused to the C-terminal end. For the gRNA cassettes in these vectors, the *AtU6-1* promoter, and sgRNA, and *AtU6* 3′end were used (pEgP236-2A-gfbsd); a custom designed gRNA can be inserted into the *Bsa*I site between *AtU6-1* promoter and sgRNA. gRNAs without off-targets were selected by CasOT *in silico* analysis^9^.

### Production and confirmation of apple transformants

For each of the four constructs, from a total of 1240–1360 leaf discs, around 40 kanamycin-resistant T_0_ apple plants were regenerated. To check for the presence of the gRNA and *Cas9* gene, as well as the *NPTII* gene, PCR analysis was performed using primers recognizing both ends of the gRNA expression cassette, the internal region of *Cas9* and the *NPTII* gene, respectively. Some regenerated plants lacking a full-length gRNA expression cassette were detected, and these were not used for further analysis (data not shown). Finally, transformants harboring sgRNA and *Cas9* as well as the *NPTII* gene were regenerated from 30–54 leaf discs for each construct (Table 1).

### Detection of mutation

For mutation analysis of each target region, primer pairs that amplify a DNA fragment of approximately 200–500 bp surrounding each target were designed based on the apple *PDS* genome sequences from ‘JM2’ and apple ‘Golden Delicious’ draft genome^1^, since the ex3–20 bp target was on the edge of the ‘JM2’ *PDS* genome sequence obtained in this study. For ex3–20 bp analysis, PDS-404F: 5′-GTTGTGATTGCTGGTGCAGGTG-3′, PDS-860R: 5′-CAATCCTTCCCTTGCTCTCCTAC-3′ were used. For ex6–20 bp and ex6–18 bp analysis, PDS-3303F: 5′-GAATATGGGCCATATTGAAGAAC-3′, PDS-3650R: 5′-CCTTGGCACAGTTCATGTTG-3′ were used, and for ex7–20 bp#49A and ex7-20bp#72B, two primer sets were used, one corresponding to the allele shown in red in Fig. 3A; PDS-4561F-26bp: 5′-tatgatttgtccttttctcgcagGGC-3′ and PDS-4704R-27bp(A): 5′-atatacCTGGAGGAATCGGTTCAAAGC-3′, and the other corresponding to the allele shown in blue in Fig. 3A; PDS-4561F-26bp: 5′-tatgatttgtccttttctcgcagGGC-3′ and PDS-4704R-27bp(G): 5′-atatacCTGGAGGAACCGGTTCAAAGC-3′ (Supplementary Fig. S1). For ex7–20 bp#89A, ex7–20 bp#49C and ex7–20 bp#70B, PDS-4321F: 5′-CTGAGAGATTGCGACAGAATACT-3′ and PDS-4823R: 5′-GCATTAATGGTGCACATGAAGC-3′ were used. The endogenous apple *PDS* genome fragments were amplified by PCR using genomic DNA from ‘JM2’. The PCR products were sequenced directly by the Sanger method or cloned into the pUC118 vector (Takara, Japan). All sequencing results were compared with the reference sequence of the wild type apple *PDS* gene by alignment in CLC main workbench 7 (Qiagen, Germany).

### Thermal Asymmetric Interlaced PCR

The T-DNA insertion sites in the transgenic apple plants were identified by TAIL-PCR using forked adapters^29^. First, the DNA was digested by restriction enzymes *Ase*I and *Rsa*I (New England BioLabs), respectively, and then ligated to the forked adapter suitable for the protrusion and blunt end, respectively. The forked adapters were as follows: 33mer_adapter: 5′-AATAGGGCTCGAGCGGCAGCTATTAATAGTACT-3′ (the last C-T was phosphorothioated), *Ase*I adapter: 5′-TAAGTACTATTAATAGCATCTTCGTTCGTCGAT-3′ (the 5′ end was phosphorylated), *Rsa*I adapter: 5′-AGTACTATTAATAGCATCTTCGTTCGTCGAT-3′ (the 5′ end was phosphorylated), the 33 mer_adapter was annealed with the *Ase*I adapter and *Rsa*I adapter, respectively. For the first PCR, the primer specific to the adapter AP2: 5′-AATAGGGCTCGAGCGGC-3′ and the primer specific to vector pEgP226-2A-gfbsdR387: 5′-TCGTGGTGGAACTAAAACAATGACC-3′ were used. For the second PCR, primers specific to adapter AP3: 5′-CGAGCGGCAGCTATTAATAGTACT-3′ and specific to vector pEgP226-2A-gfbsdR322: 5′-GGAATTTTGAGATTTCTCCCACGG-3′ were used. The amplified bands were purified and directly sequenced by using AP2 and pEgP226-2A-gfbsdR322 primers.

## Additional Information

**How to cite this article**: Nishitani, C. *et al.* Efficient Genome Editing in Apple Using a CRISPR/Cas9 system. *Sci. Rep.* **6**, 31481; doi: 10.1038/srep31481 (2016).

## Supplementary Material

Supplementary Information

## Figures and Tables

**Table 1:** Summary of mutation types of transformants targeted on the apple *PDS* gene.

phenotype	ex3–20 bp	ex6–20 bp	ex6–18 bp	ex7–20 bp
clear albino*	0	0	1	6
variegated transformants**	1	0	0	8
green transformants***	0:53	0:30	1:34	1:29
total leaf discs****	54	30	36	44

^*^Leaf disc numbers generated clear albino transformants.

^**^Leaf disc numbers generated at least one pale green or variegated transformants but no albino.

^***^Leaf disc numbers generated only green transformants with : without mutations.

^****^Leaf disc numbers generated transformants.
