# Supplementary material for: Efficient Genome Editing in Apple Using a CRISPR/Cas9 system
Source: Sci Rep. 2016 Aug 17;6:31481. doi: 10.1038/srep31481 (PMC4987624; doi:10.1038/srep31481)
Supplement: Supplementary Information [file srep31481-s1.doc]

**Title:**

Efficient Genome Editing in Apple Using a CRISPR/Cas9 system

**Corresponding author:**

Yuriko Osakabe

Faculty of Bioscience and Bioindustry, The University of Tokushima, 2-1 Josanjima, Tokushima 770-8503, Japan

Tel: +81-88-656-9310

**osakabe.yuriko@tokushima-u.ac.jp**

**Authors:**

Chikako Nishitani^1^, Narumi Hirai^1^, Sadao Komori^2^, Masato Wada^3^, Kazuma Okada^3^, Keishi Osakabe^4^, Toshiya Yamamoto^1^, Yuriko Osakabe^4^

**Authors address:**

^1^NARO Institute of Fruit Tree Science, 2-1 Fujimoto, Tsukuba, Ibaraki 305-8605, Japan

^2^Faculty of Agriculture, Iwate University, 3-18-8 Ueda, Morioka, Iwate 020-8550, Japan

^3^NARO Institute of Fruit Tree Science, 92-24, Nabeyashiki, Shimokuriyagawa, Morioka, Iwate 020-0123, Japan

^4^Faculty of Bioscience and Bioindustry, The University of Tokushima, 3-18-15 Kuramoto-cho, Tokushima 770-8503, Japan

**a 7th exon**

**
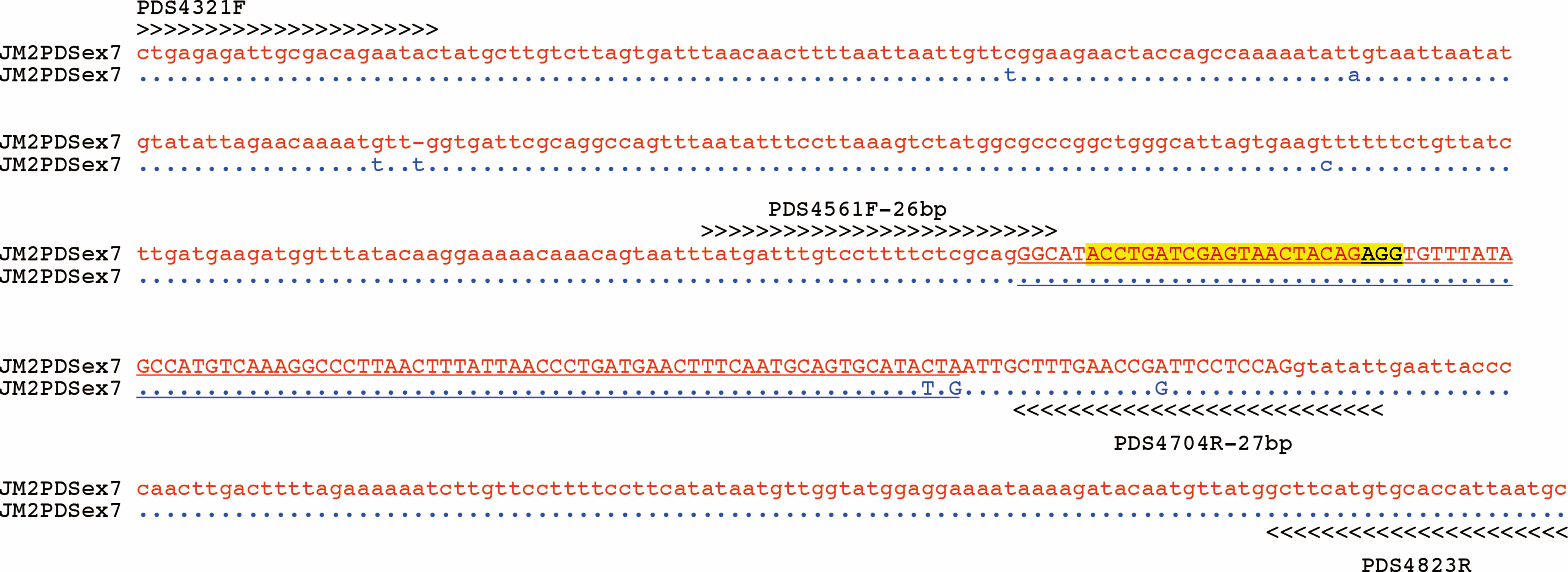
**

**b 3rd exon**

**
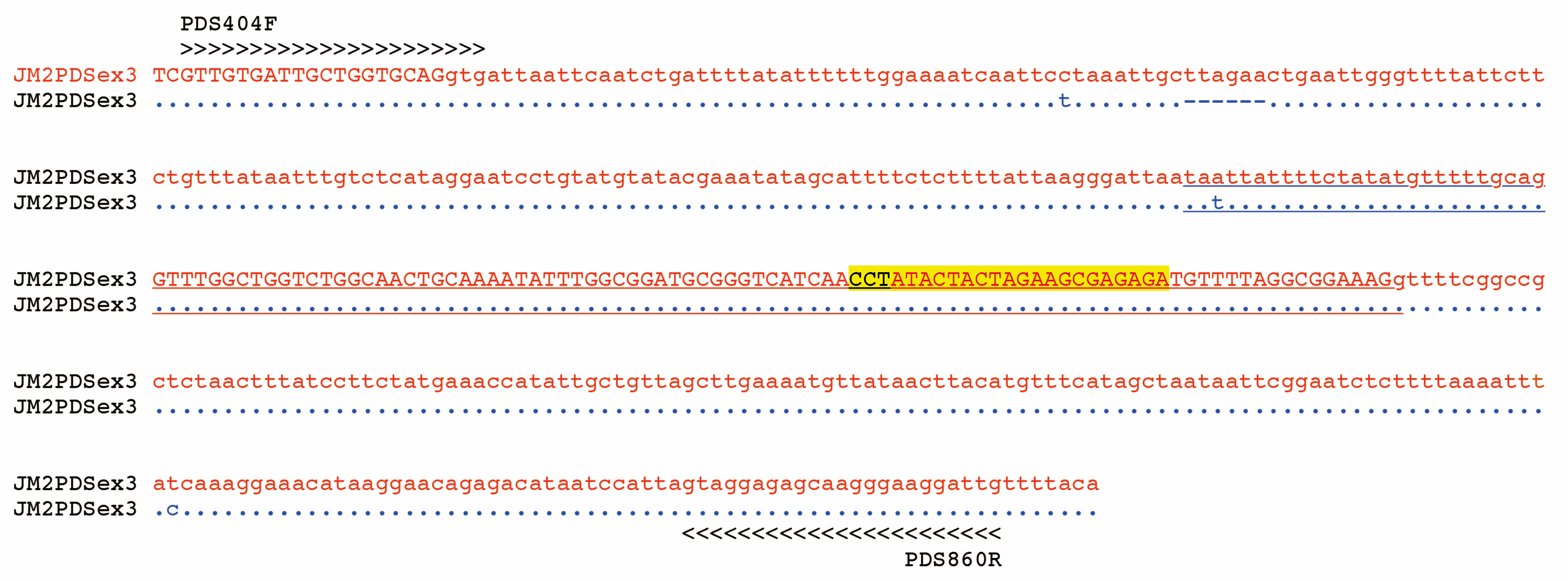
**

**c 6th exon**

**
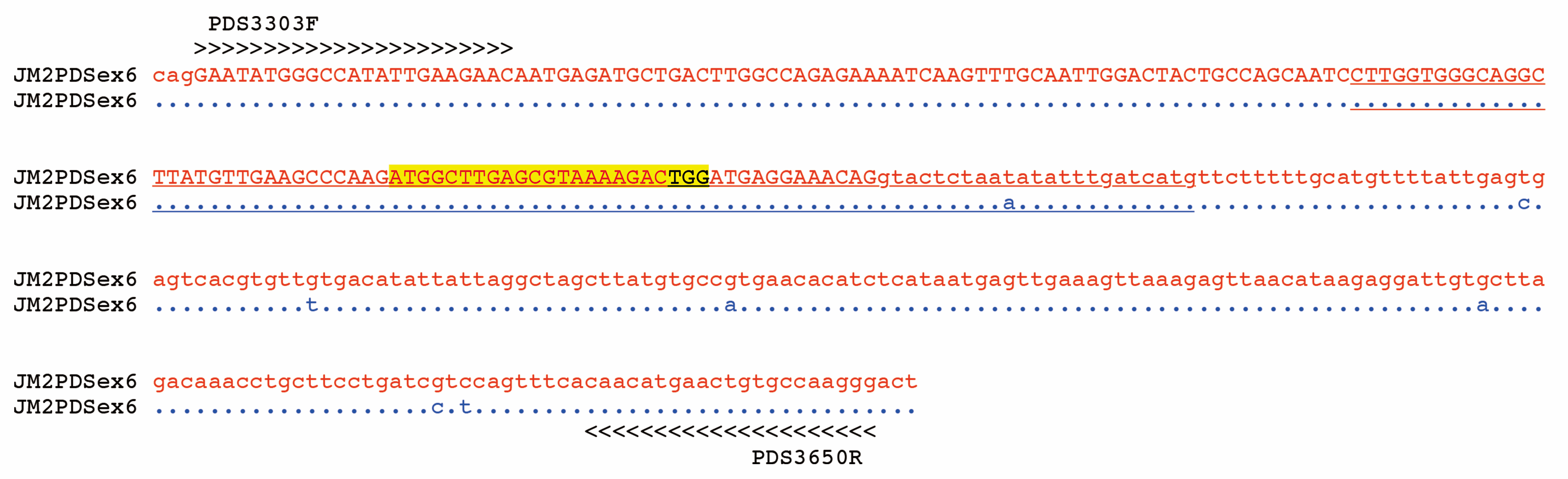
**

Supplementary Fig. S1. Alignment of two allele sequences (red and blue) around the CRISPR/Cas9 target sites in exon 7 (a), exon 3 (b), and exon 6 (c) of the apple *PDS* gene. The PCR primers used to detect the mutations are indicated. The regions shown in Fig. 3 are underlined. Several SNPs and indel sites were found in these regions. The target sites with no SNPs were indicated as yellow boxes (PAM; bold).


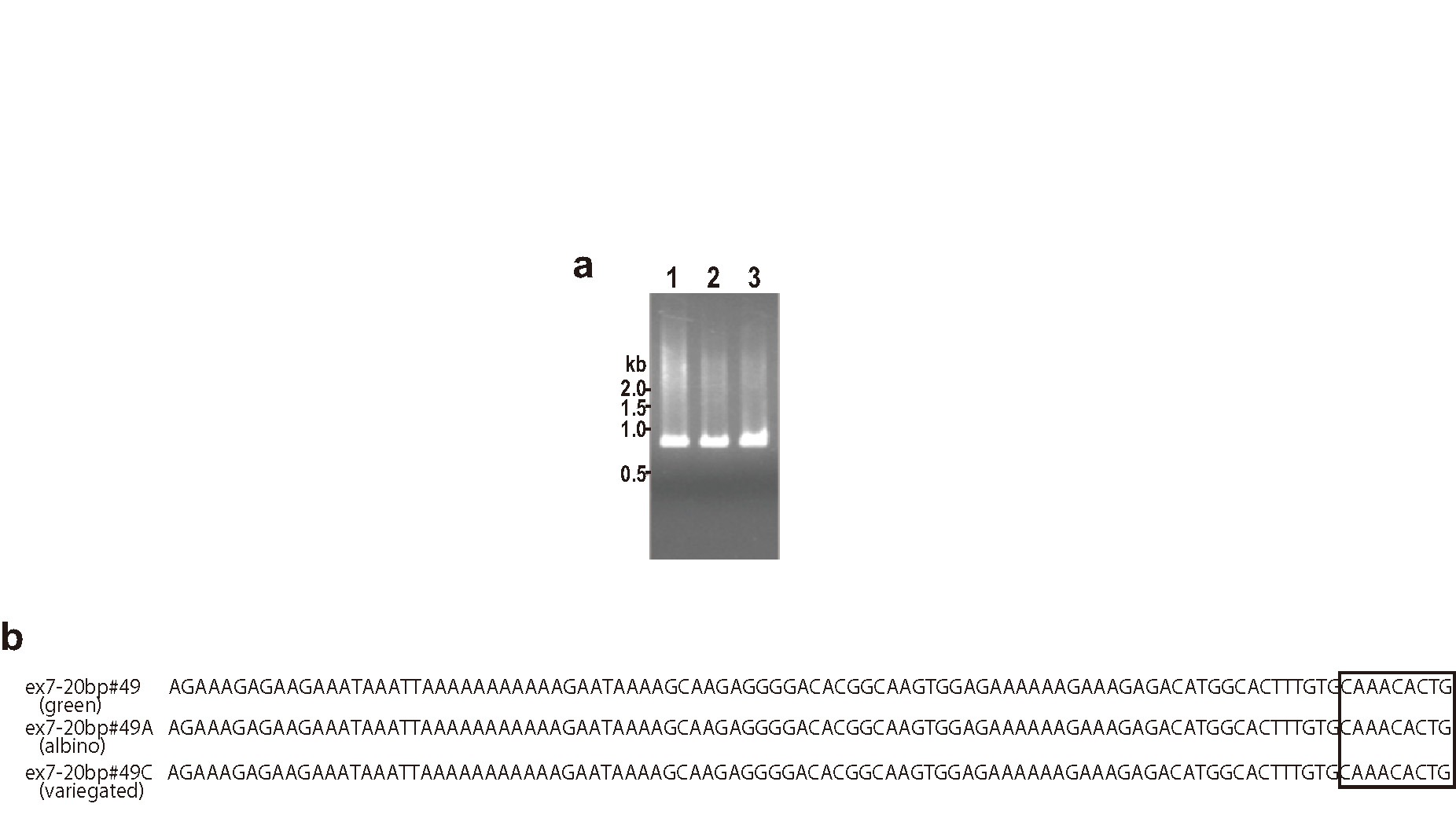


Supplementary Fig. 2. Example of T-DNA insertion sites of three apple shoots regenerated from the same leaf disc. To identify the T-DNA insertion sites, the T-DNA insertion sites in the independent shoots were amplified by tailed PCR, and directly sequenced.

a. In three independent shoots, single bands were detected in the PCR products using *Rsa*I-digested DNA. Similar results were detected using *Ase*I-digested DNA (data not shown), suggesting that a single T-DNA was introduced into each of them. 1; ex7-20bp#49, 2; ex7-20bp#49A, 3; ex7-20bp#49C.

b. The border of the apple genome and T-DNA. The predicted border sequences of the apple genome in the transgenic plants showed the same sequence, suggesting that they shared the same T-DNA insertion sites. The border sequences are identical with the apple genome (MDC013567.370 chr14:22573249..22604730, e-value=5e-35). The sequence of pEgP226-2A-gfbsd2 near the right border is boxed. ex7-20bp#49A and #49C were the plants with the visible phenotype (Fig. 2, 3), and in the ex7-20bp#49 (green shoot), no mutation was detected by direct sequence analysis.
